# Different *Cannabis sativa* Extraction Methods Result in Different Biological Activities against a Colon Cancer Cell Line and Healthy Colon Cells

**DOI:** 10.3390/plants10030566

**Published:** 2021-03-17

**Authors:** Jan Rožanc, Petra Kotnik, Marko Milojević, Lidija Gradišnik, Maša Knez Hrnčič, Željko Knez, Uroš Maver

**Affiliations:** 1 Faculty of Medicine, Institute of Biomedical Sciences, University of Maribor, Taborska ulica 8, SI-2000 Maribor, Slovenia; marko.milojevic1@um.si (M.M.); lidija.gradisnik@um.si (L.G.); 2 BioCore Institute, Nad izviri 8, SI-2204 Miklavž na Dravskem Polju, Slovenia; 3 Department of Chemistry, Faculty of Medicine, University of Maribor, Taborska ulica 8, SI-2000 Maribor, Slovenia; petra.kotnik@um.si (P.K.); masa.knez@um.si (M.K.H.); zeljko.knez@um.si (Ž.K.); 4 Laboratory for Separation Processes and Product Design, Faculty of Chemistry and Chemical Engineering, University of Maribor, Smetanova ulica 17, SI-2000 Maribor, Slovenia; 5 Department of Pharmacology, Faculty of Medicine, University of Maribor, Taborska ulica 8, SI-2000 Maribor, Slovenia

**Keywords:** extractions, *Cannabis sativa*, LC/MS-MS, cannabinoids, antioxidant, total phenols, cancer

## Abstract

*Cannabis sativa* is one of the oldest medicinal plants used by humans, containing hundreds of bioactive compounds. The biological effects and interplay of these compounds are far from fully understood, although the plant’s therapeutic effects are beyond doubt. Extraction methods for these compounds are becoming an integral part of modern *Cannabis*-based medicine. Still, little is known about how different methods affect the final composition of *Cannabis* extracts and thus, their therapeutic effects. In this study, different extraction methods were tested, namely maceration, Soxhlet, ultrasound-assisted extraction (UAE), and supercritical CO_2_ extraction methods. The obtained extracts were evaluated for their cannabinoid content, antioxidant properties, and in vitro bioactivity on human colon cancer and healthy colon cells. Our data suggest that *Cannabis* extracts, when properly prepared, can significantly decrease cancer cell viability while protecting healthy cells from cytotoxic effects. However, post-processing of extracts poses a significant limitation in predicting therapeutic response based on the composition of the crude extract, as it affects not only the actual amounts of the respective cannabinoids but also their relative ratio to the primary extracts. These effects must be carefully considered in the future preparations of new therapeutic extracts.

## 1. Introduction

*Cannabis sativa* is an annual herbaceous plant native to Central Asia but cultivated worldwide for its remarkable and broad uses in fibre and oil production, food, cosmetics, recreation, religious and spiritual moods, and medicine. The plant contains more than 500 bioactive compounds such as cannabinoids, terpenes, flavonoids, and other phenolic compounds that can be extracted from the plant to exploit their beneficial effects [1]. As a result, *Cannabis* extraction has become an important area in the modern *Cannabis*-related industry, with cannabinoid and terpene extracts becoming an integral part of the increasingly important medical, pharmaceutical, food, and cosmetic *Cannabis* sectors.

The current state-of-the-art in applying techniques for the extraction and analysis of bioactive compounds from *Cannabis*, together with research into their physical and (bio)chemical properties, has raised questions about the optimal choice of extraction methodology for recovering targeted cannabinoids as well as their specific applications. The most commonly used extraction methods include conventional solid–liquid methods such as maceration or solvent extraction, as well as a wide range of novel techniques such as ultrasound-assisted (UAE), microwave-assisted (MAE), pressurised liquid extraction (PLE), and supercritical fluid extraction (SFE) [2]. Each method requires an appropriate selection of specific conditions, such as the type of solvent, temperature, pressure, and extraction time. Since the chemical composition of *Cannabis* extracts, oils, and preparations depends on the extraction method and conditions used, great efforts have been made to optimise these to either achieve improved extraction yields or to extract the desired target compositions. Various combinations of these techniques have also been evaluated to improve the targeted recovery of specific extract compositions [3].

The discovery of the endocannabinoid system 30 years ago led to increased attention to its role in homeostasis and various diseases [4]. Considering our still relatively poor understanding of its physiological and pharmacological role in the body, targeted stimulations in in vitro models and other systematic research studies have become increasingly important. Recently, great efforts have been made to extract, identify, isolate, and quantify specific phytocannabinoids that, due to their versatility, could greatly enhance studies of targeted structure–activity relationships and thus improve our knowledge of pharmacodynamics related to specific cannabinoid receptors [5,6]. To date, more than 144 phytocannabinoids have been identified [5], with recent findings pointing to their diverse pharmacological and biological properties with the ability to act on multiple targets [7]. Such studies have already resulted in some approved cannabinoid-based medications. These include antiemetics for the treatment of nausea associated with cancer chemotherapy, formulations for the treatment of seizures associated with Lennox-Gastaut syndrome or Dravet syndrome [8,9], and medications for the treatment of anorexia associated with weight loss in acquired immunodeficiency syndrome (AIDS) patients [10]. Extensive research is currently being conducted on the potential use of *Cannabis*-based medicines to treat cancer [11,12], neuropathic pain [13,14], multiple sclerosis [15], bladder disorders [16], and more [17]. Despite the successes mentioned above with selected phytocannabinoids, there is growing evidence that either as-yet-unidentified phytocannabinoids or, more likely, combinations of cannabinoids may be more effective in treating the conditions in question [18]. Similar studies have shown that isolated cannabinoids such as Δ9-THC (Δ9-tetrahydrocannabinol) or CBD (cannabidiol) alone are less effective than more complex combinations of cannabinoids such as those found in plant extracts [19,20]. These results suggest the beneficial and, so far, only partially exploited medicinal use of *Cannabis* extracts as a complex mixture of cannabinoids, terpenes, and other molecules.

In addition to cannabinoids, various phenols (including polyphenols) present another relevant group of compounds in *Cannabis sativa*, such as prenylated flavonoids, phenolamides, and lignanamides, which are specific metabolites of this plant [21]. They are known for their multifunctional role in plants’ defence mechanism, especially through their activity as antioxidants that prevent formation of reactive oxygen species (ROS). In humans, (poly)phenols may show health-promoting effects based on the modulation of various enzymes such as lipoxygenase and the cytochrome P450 system, with cardio- or chemoprotective effects, among others [22].

Numerous pharmacological studies have shown that medicinal *Cannabis* strains exert antitumor effects both in vitro and in vivo [23,24,25]. Furthermore, specific cannabinoids such as THC was shown to induce cancer cell apoptosis and cytotoxicity by binding to cannabis receptor 1 (CB1) and cannabis receptor 2 (CB2) [26,27,28]. However, due to legal restrictions on the psychoactive cannabinoid THC, the availability and use of medicinal *Cannabis* is still limited in many parts of the world. Therefore, the present study was designed to test widely available industrial *Cannabis* or “hemp” with (known) high CBD and low THC content for its bioactive properties and to optimise extraction approaches to isolate some other known cannabinoids. In this study, the strain used was *Cannabis sativa* L. Eco-Fedora 17, a French industrial hemp plant that has been successfully cultivated for some time throughout the European Union. This variety is mainly used to produce hemp fibers, oil, and CBD, as it can reach relatively high levels of this cannabinoid. We believe that such *Cannabis* strains could also be of great use for various biomedical applications, including cancer treatment, if the right extraction method is chosen to extract the target molecules optimally. To this end, we tested different extraction methods on Eko-Fedora 17 and further examined the extracts for their underlying cannabinoid profiles, total phenol content, and antioxidant activity. While focusing on the potential medicinal use of such extracts, we also investigated how the differences in the extracts’ chemical properties affect biological activity in colon cancer cells (efficacy testing) and healthy colon cells (safety assessment).

Crude extracts are usually present in a resinous form, which, although potentially beneficial in vivo, might significantly limit potential beneficial in vitro therapeutic effects (or even lead to local cytotoxic effects due to overdosing when in direct contact with cells). A commonly used approach to reformulate prepared extracts for use in in vitro testing on cells is to dissolve them in dimethyl sulfoxide (DMSO) [29]. Although this makes the extracts applicable in cell testing, it may also affect the “primary” properties of the extracts, including the specific compositions due to a complex set of characteristics, including variable respective cannabinoid solubilities (Supplementary Materials, Table S1); [30,31,32].

In this study, we aimed to test different extraction methods and the resulting extracts for their potential biological activity in colon cancer and healthy colon cells. We also wanted to understand the limitations of post-processing of the extracts on the predictability of the final biological activity based on the crude extracts’ composition. Since such post-processing affects not only the actual amounts of the respective cannabinoids, but also their ratios compared to the crude extracts, these effects need to be taken into account when preparing novel extracts and planning their bioactivity measurement.

## 2. Results

### 2.1. Extraction Yields and Cannabinoid Composition in Crude Extracts of Cannabis sativa Obtained with Different Extraction Methods

Due to their known high extraction yields (EY), dynamic maceration and Soxhlet extraction were selected to compare their effectiveness with modern extraction methods such as UAE and SFE. The selected extraction methods were optimised to maximise the cannabinoid content in the extracts. In addition to the common cannabinoids, CBD, THC, and their acid forms, three other cannabinoids (i.e., cannabichromene (CBC), cannabinol (CBN), and cannabigerolic acid (CBGA)) were selected for analysis. The latter were selected due to their importance in phytocannabinoid biosynthesis [33] and their bioactive properties [34]. Since all the chosen extraction methods also lead to the extraction of a certain fraction of phenols known to possess antioxidative properties, these were also evaluated.

**Figure 1:**
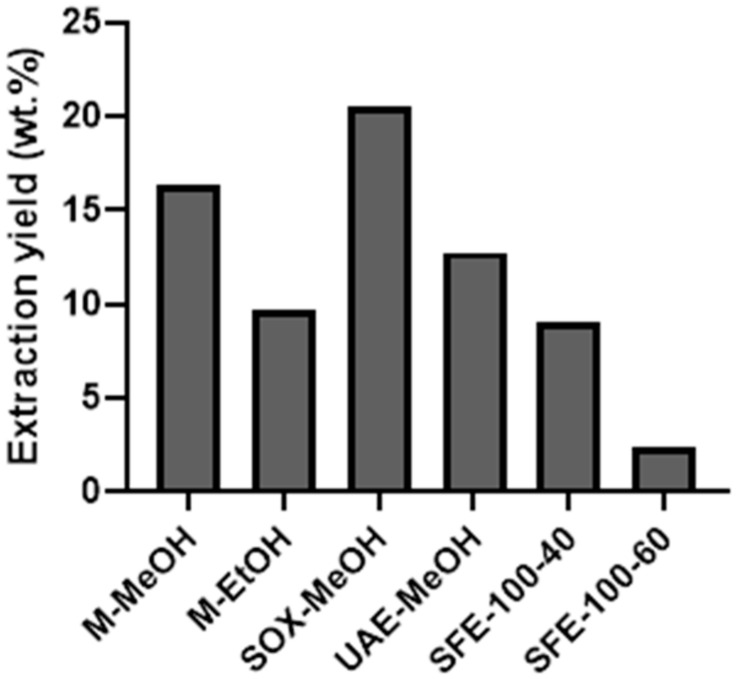
Extraction yield (EY) with different extraction methods (maceration with methanol—M-MeOH, maceration with ethanol—M-EtOH, Soxhlet extraction with methanol—SOX-MeOH, ultrasound-assisted extraction—UAE, supercritical CO_2_ extraction at 100 bar and 40 °C—SFE-100-40, supercritical CO_2_ extraction at 100 bar and 60 °C—SFE-100-60). Data are expressed as weight percentage (wt %) of the extracts obtained per 100 g of dry flower material.

During extraction optimisation, the alcohols methanol and ethanol were found to be the most suitable extraction solvents for cannabinoid isolation in “conventional” extraction methods. They were therefore chosen for maceration and Soxhlet extraction. It should be noted that in the literature, supercritical CO_2_ is also reported among one of the most effective cannabinoid solvents as is also the use of hexane [35,36,37,38]. The conventional extraction methods gave similar extraction yields to supercritical fluid extraction with CO_2_ at temperatures of 40 and 60 °C and a pressure of 100 bar. The extraction yields (EY) of the optimised methods are shown in Figure 1. The highest EY of 20.5 wt % was obtained by Soxhlet extraction, followed by maceration in MeOH with 16.3 wt %. EtOH was observed to be less effective in maceration, which is in agreement with a study published by Rovetto et al. [36]. The ethanol extraction reported in this study gave an EY of 13.2 wt %. The same authors also reported a relatively low EY for their SFE method of 7.4 wt % (for this, they used a low incremental pressure adjustment with the maximum pressure of 340 bar and a temperature of 55 °C). Although higher pressures (~300 and 400 bar) are common in SFE (in industrial settings, while they are less common on a laboratory scale), they were not applicable in our study as we wanted to avoid unnecessary extraction of fatty components and chlorophylls. Namely, the latter are known to be successfully extracted under the mentioned conditions [37]. None of the methods included an (additional) decarboxylation step to preserve the acid forms in their natural state.

Quantitative analysis of cannabinoids in the crude extracts was performed by high-performance liquid chromatography (HPLC) analysis in tandem with mass spectrometry (MS/MS). A representative LC-MS/MS chromatogram of a *Cannabis* extract sample is shown in the Supplementary Materials (Figure S1). The cannabinoids were identified and quantified based on ion transitions, and the results are shown in Table 1. Since the plant material used in this experiment was the industrial *Cannabis* strain Eco-Fedora 17, it is not surprising that CBD and CBDA are the most abundant cannabinoids [39]. Based on the results obtained, several conclusions can be drawn. The first observation shows that the total CBD content is similar for all methods used, suggesting that all extraction methods have similar efficiency in terms of CBD extraction. However, important differences could be observed regarding the respective CBD and CBDA contents, which require further consideration and analysis. First, one has to consider the solubilities of the respective cannabinoids in the extraction solvents used (MeOH, EtOH, and SF CO_2_). Unlike CBD, CBDA has a higher polarity due to lack of the carboxylic acid group and is, therefore, more soluble in polar solvents. Consequently, it is more likely to be better extracted in polar solvents. Similar trends are known for other cannabinoids, for whose acidic counterparts are better extracted in polar solvents. Another important aspect related to the ratio between cannabinoids and “their acids” is the conversion rate, which is also known to vary between different extraction methods [40]. A general observation is that higher energies (either in the form of heat, UV light, or ultrasound) promote decarboxylation. The highest amount of extracted CBD was obtained using Soxhlet and MeOH. In contrast, the highest CBDA levels were obtained by SFE (similar amounts could be extracted regardless of the conditions used), which is consistent with the literature [35]. These results agree quite well with other literature sources. Namely, we know that such highly energetic types of extractions (e.g., using Soxhlet extraction) can yield higher amounts of decarboxylated cannabinoids [41].

Considering the extraction of THC and THCA, it is immediately clear that the industrial *Cannabis* used does not possess very large amounts of these cannabinoids. Yet, there are still important differences between their capability to extract THC and its acidic counterpart. The highest total amount of extracted THC was retrieved with EtOH maceration, but differences in the extracted amounts of THC and THCA were also found between the other methods used. SFE extraction at 100 bar and 40 °C presents the only method that results in the extraction of more THCA than THC. It is worth noting that MeOH maceration and SFE at 100 bar and 60 °C resulted in similar total THC values. In contrast, the former method extracted mainly THC, while the latter resulted in the extraction of a ratio of approximately 3/1 of the mentioned cannabinoids.

**Figure 2:**
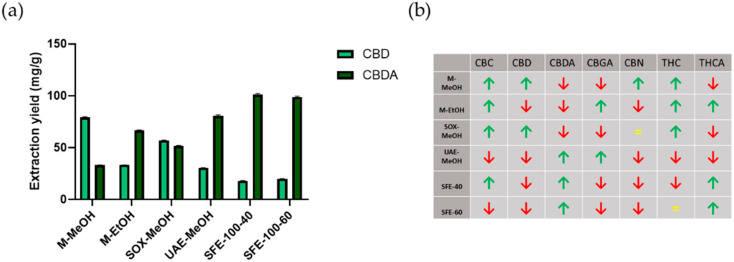
Comparison of cannabinoid extraction yields with different extraction methods. Panel (**a**) shows the amounts of CBD and CBDA obtained with different extraction methods (data are means ± SD from n = 3 replicate measurements), and panel (**b**) shows the trends of all cannabinoids analysed across extraction methods tested compared to the average (= means as the average, ↑ above average, and ↓ below average).

Finally, as mentioned above, although an industrial *Cannabis* strain was used, we still wanted to evaluate the potential use of these extraction methods to extract other, less abundant cannabinoids. As shown in Table 1 (and graphically depicted in Figure 2), significant differences occur in the extraction of the cannabinoids CBC, CBGA, and CBN. This suggests that great care must be taken to choose the extraction method to allow fine-tuning of extract compositions based on desired pharmacological effects [42]. On the other hand, we must not forget that we are only at the beginning of understanding the structure-activity relationships of the different cannabinoids, especially with regard to the already observed and reported entourage effect [18,20,43]. These results (content variability) indicate a great promise that such formulation variations could already be achieved by applying different extraction methods, without the need for respective cannabinoid isolations and their mixing in desired combinations. Looking at the concrete contents obtained, we see that maceration with MeOH leads to the highest extracted amounts of CBC, while maceration with EtOH leads to the highest amounts of CBGA. In contrast, CBN content differences are the smallest, indicating that neither method is suitable for its extraction [44] or that the used *Cannabis* strain actually contains little of this cannabinoid. It is also known from the literature that higher amounts of CBN can “only” be obtained by decarboxylation of *Cannabis* extracts. For easier observation of the obtained compositions using different extraction methods, we created a visual representation of the extracted amounts based on the calculated average (Figure 2b).

### 2.2. Total Phenol Content and Antioxidant Activity of Cannabis Extracts

The total phenolic content and antioxidant activity of the crude *Cannabis* extracts were also investigated (the results are presented in Table 2). The different extraction methods used gave a wide range of total phenolic concentrations, ranging from 38.3 to 145.9 of the gallic acid equivalent (GAE). Maceration with EtOH resulted in a significantly higher yield of total phenols than MeOH (*p* = 0.000064), but no significant changes were observed compared to the Soxhlet-EtOH method (*p* = 0.78). Compared with the lowest yield of total phenols obtained by the SFE method, which is in agreement with the literature [45,46], the ultrasound-assisted extraction gave the highest yield (145.9 GAE), which is also in agreement with previous studies [47,48]. Moreover, the antioxidative properties measured by the DPPH assay correlated highly with the total phenolic content (*p* = 0.005) [49,50]. It should also be noted that, except for the two SFE methods, all other extracts exhibit a relatively high antioxidant activity. This could indicate their potential use as dietary supplements, either in general or as supplements in cancer treatment, where many chemotherapeutic agents produce ROS and, therefore, could damage healthy cells [51]. Full data evaluating statistically significant differences between the samples are shown in Supplementary Materials, Table S2 for the total phenolic content, and Table S3 for the DPPH measurements.

### 2.3. Preparation (Post-Processing) of Cannabis sativa Extracts for Cell Culture Treatment

In general, cannabinoids are poorly water-soluble but highly soluble in lipids. Because of their poor water-solubility, they cannot be used directly to treat cells and measure their potential pharmacological effects. A common approach of their post-processing to enable such testing is to dissolve them in DMSO. By doing this, we cannot avoid the fact that this dissolution may alter the composition of crude extracts. Effects governing the composition of the resulting “secondary” extracts (DMSO solutions) are multifold. Probably the greatest influence on this composition relates to the respective solubility of the cannabinoids in DMSO (Supplementary Materials, Table S1). However, other, more complex effects need to be considered. These include possible complexation during dissolution, conversion of cannabinoids to their acid forms, and other effects [51,52].

**Figure 3:**
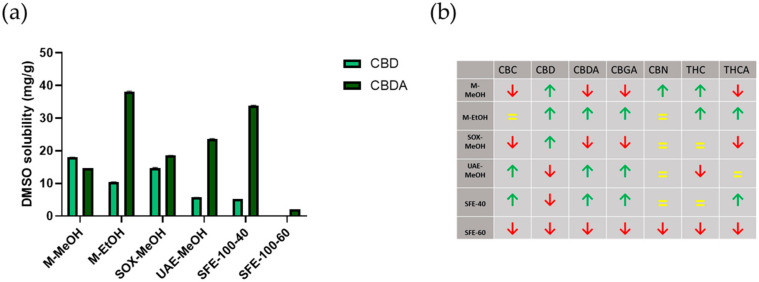
Comparison of cannabinoid content in *Cannabis sativa* extracts dissolved in DMSO. Panel (**a**) shows CBD and CBDA solubility in DMSO between different extraction methods (data are means ± SD from n = 3 replicate measurements). Panel (**b**) shows the trends of all cannabinoids dissolved in DMSO across extraction methods tested compared to the average (= means as the average, ↑ above average, and ↓ below average).

Again, quantitative analysis of cannabinoids in the crude extracts was performed by HPLC analysis in tandem with mass spectrometry (MS/MS). The cannabinoids were identified and quantified based on ion transitions, and the results are shown in Table 3 (and graphically drawn in Figure 3). For easier observation of the obtained compositions after the dissolution of the crude extracts in DMSO, we again created a visual representation of the extracted amounts based on the calculated average (Figure 3b). Our initial idea were that the resulting compositions would correlate, for the most part, with the input composition of crude extracts and the respective solubility of the cannabinoids. Statistical analysis of phytocannabinoid content identified significant differences between crude extracts and DMSO dissolved extracts (Supplementary Materials, Table S8). Furthermore, we performed a statistical analysis regarding the possible correlation between the concentrations of the respective cannabinoids in the crude extracts, their solubilities, and their concentrations in the DMSO solution (not shown). No such correlation was found. These results indicate that more complex yet unknown effects are present in such complex extracts, and further analysis would be necessary to fully understand the phenomena governing such processes [45,46].

### 2.4. Effect of Cannabis sativa on Colon Cancer Cell Survival

**Figure 4:**
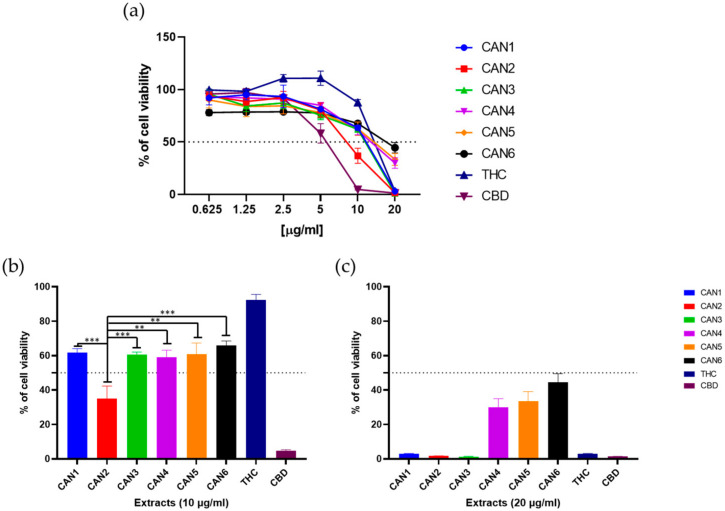
Responsiveness of the Caco-2 cell line to different *Cannabis sativa* extracts, THC, and CBD. (**a**) Cells were treated with different concentrations (0.625–20 µg/mL) of the extracts for 48 h, and the MTT assay determined cell viability. Sections (**b**,**c**) show the concentrations of 10 µg/mL and 20 µg/mL, respectively. Data show cell viability relative to untreated controls (mean ± S.D. from n = 4 repeated measurements). Statistical significance is defined as ** *p* < 0.005 and *** *p* < 0.0005 (ANOVA test) for all sample comparisons.

Experiments to analyse cell viability were performed on the human colon carcinoma cell line (Caco-2). Cells were treated with increasing concentrations (0.6–20 µg/mL) of *Cannabis* extracts (CAN1–CAN6) and pure THC and CBD for 48 h. The MTT assay was used to determine cell survival/viability. The effects of these 6 extracts and 2 pure cannabinoids on the survival of the Caco-2 cell line are shown in Figure 4a. As shown, all extracts and pure cannabinoids affected Caco-2 cell survival in a dose-dependent manner.

Moreover, M-EtOH extract (CAN2) exhibited the most potent anticancer properties and significantly outperformed the other extracts at 10 ug/mL (Figure 4b) with an IC_50_ value of 8.63 µg/mL (Table 4. In contrast, CAN4–CAN6 showed the lowest potency, achieving only 30–45% of cell viability at 20 µg/mL (Figure 2b). Interestingly, pure CBD showed potent anticancer properties with an IC_50_ value of 6.06 µg/mL, while pure THC increased the cell viability of Caco-2 cells at low micromolar concentration. Full data evaluating statistically significant differences between the samples are shown in Supplementary Materials, Tables S4 and S5.

### 2.5. Stimulatory Effects of Cannabis sativa Extracts on Untransformed Intestinal Cells

**Figure 5:**
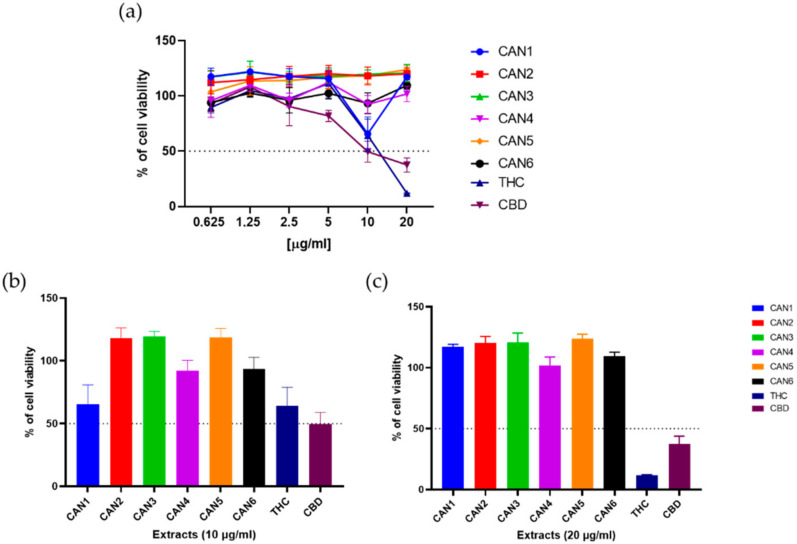
Responsiveness of HUIEC cells to different *Cannabis sativa* extracts, THC and CBD. (**a**) Cells were treated with different concentrations (0.625–20 µg/mL) of the extracts for 48 h, and cell viability was determined by MTT assay. Sections (**b**,**c**) show the concentrations of 10 µg/mL and 20 µg/mL, respectively. Data show cell viability relative to untreated controls (mean + S.D. from n = 4 replicate measurements).

When normal human intestinal epithelial cells (HUIEC, isolated in our lab [53]) were treated under the same experimental conditions, most extracts showed stimulatory effects by increasing cell viability by up to 25% compared to untreated controls (Figure 5). While only one extract (CAN1) at 10 µg/mL resulted in a modest decrease in cell viability (Figure 5b), the general trend suggests stimulatory effects of *Cannabis* extracts on cell viability in non-transformed intestinal cells.

In contrast, a significant decrease in cell viability was observed when pure THC and CBD were used, resulting in a loss of cell viability to 11.7% and 37,5%, respectively, at 20 µg/mL (Figure 5c). Full data evaluating statistically significant differences between the samples are shown in Supplementary Materials, Tables S7 and S8.

## 3. Discussion

In the present study, four extraction methods (with varying parameters, a total of 6 extracts were prepared) were performed to obtain *Cannabis* extracts with variable cannabinoid content. These were tested for their antioxidant properties and evaluated for their in vitro biological activity on human colon cancer and normal colon cells. The bioactivity of pure THC and CBD was studied for comparison.

The extracts were analysed by LC-MS/MS to detect seven cannabinoids (CBD, CBDA, THC, THCA, CBGA, CBC and CBN). As expected for an industrial *Cannabis* strain, CBDA was the main cannabinoid present in all extracts (the material was not intentionally decarboxylated) [54]. Since neutral forms are more soluble in supercritical CO_2_, the extracts’ compositions showed that fresh plant material was used. The antioxidant properties and biological activity had to be screened to evaluate the different prepared extracts for their potentially variable pharmacological effects.

In addition to CBD, other cannabinoids with potentially bioactive properties have been determined. THC (and its precursor THCA) is the main psychoactive compound of the *Cannabis* plant. However, several techniques have been developed to extract other non-psychoactive compounds without the psychoactive ones [32,42]. On the other hand, the recovery of THC for its medicinal applications could be increased if optimised extraction methods are used. Our results indicate that variable extract compositions can be obtained (even tailored to some extent) by choice of extraction method and the conditions used to perform it, which agrees with recently published studies [55,56,57].

Apart from CBD and THC, we also assessed CBC, CBGA, and CBN levels, which are important molecules being investigated for various biomedical applications. For example, CBC has shown promising results for the selective reduction of inflammation-induced hypermotility in vivo [58], as well as potent anti-inflammatory activity and may lead to the amelioration of murine colitis [59]. It is worth noting that ultrasound-assisted extraction yielded significantly higher CBC levels than other methods at 1.8 mg/g because the temperature in UAE can reach up to 40 °C for 120 min, and the acid form of CBC can be decarboxylated to neutral form. The CBC acid form (CBCA) was not analysed in this study because no analytical standard could be found for it.

Overall, the M-EtOH, UAE, and SFE-40 methods were better for retaining acidic cannabinoids, which is consistent with the literature. Namely, several studies have been published indicating the importance of additional decarboxylation steps necessary to increase CBD and THC contents [40,54]. Decarboxylation can be achieved by adding energy to the system (as in the case of high temperature or UV light), while simple solvent changes (e.g., MeOH for EtOH) do not affect the latter. The same was observed in our study, where only the Soxhlet extraction, which uses a higher temperature compared to the other extractions, led to an increase in decarboxylated cannabinoids.

In addition to cannabinoid analysis, total phenolic content and antioxidant activity were evaluated and showed a high correlation, with ultrasound-assisted extraction yielding the highest amounts of total phenols extracted. Antioxidative effects are often measured in various plant extracts. The latter are commonly used as part of multi-drug regimens, where the potentially toxic effects of the main drug need to be counterbalanced. Chemotherapeutic agents with known cytotoxic effects are also one such example, where extracts could reduce the extent of adverse effects on healthy cells [51,52].

This study aimed to determine the potential of various extraction methods to tailor the extract-specific cannabinoid compositions and evaluate cannabinoid determination in crude extracts to predict their biological activity. The latter is not straightforward, as the measurement of cell-based effects (e.g., cytotoxicity) requires water-soluble extract compositions. For this purpose, the crude extracts are usually dissolved in DMSO, which may be the limiting factor in maintaining the initial extract’s composition and the ratios between the respective extracted cannabinoids, potentially affecting the entourage effect. Herein, we statistically evaluated the correlations between the initial extracts, their solutions, and respective solubilities of the cannabinoids in DMSO. Our results suggest that a direct prediction of biological activity based on crude extracts’ composition should be taken cautiously. Among the properties that must be considered to allow such predictions are the respective solubilities of the cannabinoids, although far more complex and more difficult to determine phenomena also control such dissolutions. In our case, no correlations could be found between the crude extract compositions and the respective cannabinoid solubilities to obtain cell test solutions. In general, the final extract’s effect can be presumed to be related to the most abundant cannabinoids. Simultaneously, a more complex mechanistic understanding of the effects also requires a more complex determination of the effects. Thus, this would be a future research direction to better understand the complex biological activity profiles of various *Cannabis* extracts.

The experiments performed on the colon cancer cells confirmed that *Cannabis* extracts have selectively decreased these cells’ viability, which agrees with recently published studies [25,60]. An important beneficial side effect is that they simultaneously have a stimulatory effect on healthy intestinal cells. Several studies have indicated such cancer-specific mode of action [24,61], although the exact molecular mechanisms remain an important open question in the field. As already discussed in the literature, the high antioxidative properties of *Cannabis sativa* could be the reason for the protection of healthy cells from the cytotoxic effect of various chemotherapeutics. The same effect could also counterbalance the extracts’ antiproliferative effects on healthy cells as observed in this study [62,63].

Overall, our data indicate that *Cannabis* extracts when properly prepared, could decrease cancer cell viability while protecting healthy cells from cytotoxic effects produced either by the formation of ROS or by other as-yet-unknown mechanisms. Furthermore, we have shown that variation in the extraction method can lead to different compositions of the final extract composition and thus to different biological effects. Although we are aware that several additional studies are required to refine such approaches towards targeted pharmacologic effects, we nevertheless believe that the obtained results are already promising. Further studies should also be directed towards determining the exact mechanisms of action (general and side effects), either on cannabinoid receptors (directly or indirectly) or through other target molecules’ modulation.

Finally, although one would assume that the crude extract’s composition is a good indication of the final biological effects, such assumptions require a more careful evaluation. A particular challenge relates to the fact that testing of pharmacological effects must be conducted in vitro. In such settings, the extracts cannot be used in their pure form but have to be rendered water-soluble. In this case, the resulting solutions could be significantly different from the crude extracts’ primary compositions, including the ratios between the respective present cannabinoids. This can affect not only the final biological effects of the crude extracts, but also their potential entourage effect. Therefore, we believe that future studies must include systematic evaluation methods to address this issue, leading to a better understanding of the effects of respective extract compositions.

## 4. Materials and Methods

### 4.1. Chemicals and Reagents

Standard solutions of cannabidiol (CBD), cannabidiolic acid (CBDA), Δ^9^-tetrahydrocannabinol (Δ^9^-THC), Δ^9^-tetrahydrocannabinolic acid (Δ^9^-THCA), cannabigerolic acid (CBGA), and cannabinol (CBN) and cannabichromene (CBC) were purchased from Sigma-Aldrich (Sigma-Aldrich, Germany). Formic acid (HCOOH), acetonitrile (ACN), methanol (MeOH) and ethanol (EtOH) HPLC grade were purchased from Merck (Germany). Reagents for radical scavenging activity measurement (DPPH, 2,2-diphenyl-picryl-hydrazil) and determination for phenolic content (Folin–Ciocalteu reagent, Na_2_CO_3_, gallic acid) were purchased from Sigma-Aldrich (Sigma-Aldrich, Germany).

### 4.2. Plant Material

The plant material (female inflorescences) used for extractions was provided by a local farm Makoter (Makoter, Slovenia). Industrial species *Cannabis sativa* L., Eco-Fedora 17 variety, lot number F1545 (SOC France, France) was used for extract preparation.

### 4.3. Extraction Methods

*Cannabis* extraction was performed using a Soxhlet extractor, maceration, ultrasonic extraction with different solvents (methanol, ethanol), and supercritical extraction with carbon dioxide at different temperatures (40 °C and 60 °C) and pressures of 100 bar. The extraction methods are briefly described below. Sample were denoted as shown in Table 5.

#### 4.3.1. Soxhlet Extraction

Approximately 15 g of the material was weighed into a filter bag, which was placed in the cylindrical part of the Soxhlet apparatus. Then, 180 mL of the solvent was heated to reflux. After 240 min of extraction at a temperature above the boiling point of the solvent, the solvent was removed using a vacuum rotavapor at a temperature of 40 °C. The extract was stored in a freezer at −20 °C until analyses.

#### 4.3.2. Dynamic Maceration

The ground material (15 g) was extracted by stirring with a magnetic stirrer in 180 mL of solvent for 4 h at room temperature. The mixture was filtered to remove solid particles and concentrated under vacuum at 40 °C. The extract was stored in a freezer at −20 °C until the analyses.

#### 4.3.3. Supercritical Fluid Extraction

Supercritical fluid extraction experiments were performed on an extraction unit previously described in the literature [64]. Approximately 15 g of ground material was placed in the extractor, which was heated in a water bath at a constant temperature. Pressurised CO_2_ was introduced into the gas cylinder’s extractor using a high-pressure pump and maintained at a constant flow of 2.5 mL/min throughout the experiment. The extract was collected in the sampling tube at room pressure and 15 °C. The total extraction time was 180 min. The extract was stored in a freezer at −20 °C until the analyses.

#### 4.3.4. Ultrasound-Assisted Extraction

The ultrasound-assisted extraction method has been used to isolate active ingredients from raw materials. Ultrasonic waves are used to cause cavitation in the solvent, thereby accelerating the dissolution and diffusion of solutes from the raw material. During extraction at ratio material:solvent = 1:12 (15 g of material: 180 mL of solvent), the temperature was controlled at the desired level at 40 °C within ± 1 °C. Ultrasonic irradiation was carried out for 120 min at 500 W and 50 Hz using ultra-sonic bath Iskra PIO—Sonis 4 (Iskra, Slovenia). After extraction, the mixture was filtered to remove solid particles, and the solvent was evaporated using a vacuum rotavapor. The extract was stored in a freezer at −20 °C until the analyses were performed.

Extracts were dissolved in 10 % DMSO to prepare the concentration of extracts of cca. 1 mg/mL for the determination of cannabinoid content and further bioassay studies. For this purpose, the extracts (~1 mg) were put into dark HPLC vials (Agilent, Germany), and 100 µL of DMSO was poured onto them. For the dissolution, as-prepared vials were put into a dark cabinet for 1 h. After 1 h, 900 µL of ultra-pure water was poured into the vials to form extract solutions with a concentration ~1 mg/mL.

### 4.4. LC-MS/MS Analysis

Cannabinoid content was determined by LC-MS/MS, as previously described [65]. Analyses were performed on Agilent 1200 HPLC in tandem with mass spectrometry Agilent 6460 JetStream triple quad detector. Agilent Poroshell EC-C18 column with a particle size of 2.7 µm and dimensions of 100 × 2.1 mm ID was used. The mobile phase consisted of ultra-pure water with the addition of 0.1% formic acid (A) and acetonitrile with 0.1% formic acid (B) using the gradient method with a flow of 0.3 mL/min. The gradient method was composed of multiple linear gradients as follows: 0 min, 34% B; 8 min, 34% B; 12 min, 95% B; 13 min, 95% B; 14 min, 95% B; 20 min, 34% B; and 3 min post time. Detailed parameters for LC-MS/MS method are shown in Table 6.

The column temperature was stable at 35 °C. Negative ionisation was used on the mass spectrometer, the temperature of the carrier gas was 300 °C, and that of the supporting gas was 250 °C. The total gas flow was 16 L/min.

The calibration curve for each cannabinoid was plotted in the concentration range of 2.5–200.0 ng/mL with good linearity in the measurement range between calibration points, R2 > 0.999. All points were measured in triplicates with an RSD < 3%.

### 4.5. Determination of Total Phenolic Content

The total phenolic content in the extracts was determined using the Folin–Ciocalteu reagent as described in the literature [66]. Briefly, the Folin–Ciocalteu reagent solution was prepared by diluting the basic Folin–Ciocalteu reagent solution with distilled water in a ratio of 1:10. The Na_2_CO_3_ solution was prepared by weighing approximately 3.75 g of Na_2_CO_3_ into a 50 mL volumetric flask, diluted to the mark with distilled water, and sonicated until the Na_2_CO_3_ was completely dissolved. Approximately 20 mg of the extract was weighed into a 10 mL volumetric flask and diluted with methanol. The mixture of 150 μL of the reagent solution Folin–Ciocalteu and 120 μL Na_2_CO_3_ was added to 30 μL of the prepared extract solution. The mixture was left at a temperature of 50 ± 1 °C for 5 min, then the absorbance of the solution was measured at 760 nm using a UV-visible spectrophotometer. Total phenolic compounds were determined in triplicate for each sample. The gallic acid calibration curve was used to quantify the total phenolic compounds, and the amount of phenolic compounds in the samples was expressed as gallic acid equivalents in mg gallic acid/g extract.

### 4.6. Antioxidant Assay

The radical scavenging activity of the extracts was measured using the stable radical reagent DPPH (2,2-diphenyl-picryl-hydrazil) [67]. Next, 10 μL of the extract solution (1 mg/mL) in methanol was added to 200 μL of the methanol solution of DPPH (0.025 g/L). In parallel, a negative control was prepared by mixing 10 μL of methanol with 200 μL of the methanol solution of DPPH. After 15 min incubation in the dark at room temperature, the absorbance was measured at 517 nm against a blank sample. As a positive control of an antioxidant reference, ascorbic acid was used, which was measured under the same conditions as the samples. Antioxidant activity against DPPH radicals was expressed as a percentage of inhibition.

### 4.7. Cell Cultures

Two cell lines were used for the biological activity analysis of the developed *Cannabis* extracts: Caco2 (human colorectal adenocarcinoma cells) (ATCC^®^ HTB-37™) and HU -IEC (human intestinal epithelial cells), previously isolated and characterised by our group [53]. Cells were grown in Dulbecco’s modified Eagle’s F12 medium (DMEM/F12) supplemented with 10% FBS, 1% L-glutamine, penicillin (100 U/mL) and streptomycin (1 mg/mL) at 37 °C in 5% CO_2_ atmosphere in tissue culture flasks.

### 4.8. Cell Viability Assay

To assess the effect of *Cannabis* extracts on cell viability, cells were seeded in 96-well plates containing 10,000 cells/well and allowed to adhere overnight. The following day, the cells were treated with different *Cannabis* extracts (CAN1-CAN7), and purified THC and CBD isolates at different concentrations for 48 h. The cells were then allowed to grow in the medium. After treatment, the medium was replenished, and the cells were treated with tetrazolium salt MTT [3(4,5-dimethylthiazolyl-2)-2,5-diphenyltetrazolium bromide] reagent (Sigma Aldrich, Germany) and incubated for 4 h at 37 °C. After incubation, the medium was removed, and DMSO was used to dissolve the purple formazan product, which is proportional to the viable cells [68]. Each sample’s absorbance was measured spectrophotometrically at 570 nm wavelengths using a Varioscan Flash Multiwell plate reader (Thermo Scientific, MA, USA). The percentage of cell viability was expressed relative to untreated controls. The IC_50_ value, i.e., the concentration of test samples required to inhibit 50% of cancer cell growth relative to controls, was extrapolated from a concentration-response diagram.

### 4.9. Statistical Analysis

All numerical values are given as mean ± standard deviation (SD). The Shapiro–Wilk test confirmed the normal distribution of the experimental data. Levene’s test was used to assess the equality of variances. Since all data sets were well-modelled by a normal distribution and were homoscedastic, a one-way analysis of variance (ANOVA) followed by the Bonferroni post hoc test was performed accordingly. Spearman’s correlation was used to analyse the statistical association between variables. Obtained *p*-values < 0.05 were considered statistically significant. Statistical analysis was performed using SPPS Statistics 27 (IBM Corp. Armonk, NY, USA).

## Figures and Tables

**Table 1:** Phytocannabinoids in *Cannabis sativa* extracts analysed by LC-MS/MS. Data are expressed as mg/g (mean, n = 3 ± SD). Total CBD and THC are calculated as the sum of natural form, plus acid form, multiplied by conversion factor 0.877 to account for the loss of CO_2_ molecule during the decarboxylation.

Sample	Extraction Method	CBC	CBD	CBDA	CBGA	CBN	THC	THCA	Total CBD	Total THC
CAN1	Maceration with MeOH	4.743 ± 0.210	79.107 ± 0.759	33.073 ± 0.211	1.761 ± 0.009	0.4010 ± 0.005	4.421 ± 0.111	0.452 ± 0.003	108.11	4.82
CAN2	Maceration with EtOH	3.543 ± 0.091	33.258 ± 0.112	66.597 ± 0.425	4.889 ± 0.015	0.189 ± 0.002	4.340 ± 0.98	2.023 ± 0.01	91.66	6.11
CAN3	Soxhlet with MeOH	3.908 ± 0.088	56.936 ± 0.119	51.674 ± 0.359	2.041 ± 0.066	0.311 ± 0.003	3.478 ± 0.89	0.522 ± 0.002	102.25	3.94
CAN4	UAE with MeOH	2.083 ± 0.076	30.402 ± 0.112	80.633 ± 0.891	3.284 ± 0.102	0.196 ± 0.004	3.018 ± 0.85	1.083 ± 0.009	101.12	3.97
CAN5	SFE CO_2_ at 100 bar, 40 °C	3.701 ± 0.106	18.119 ± 0.104	100.948 ± 1.26	1.760 ± 0.071	0.212 ± 0.005	0.722 ± 0.009	2.467 ± 0.009	106.65	2.89
CAN6	SFE CO_2_ at 100 bar, 60 °C	0.096 ± 0.005	19.908 ± 0.099	98.634 ± 0.775	1.223 ± 0.009	0.668 ± 0.023	3.291 ± 0.103	1.708 ± 0.008	106.41	4.79

**Table 2:** Effect of different extraction methods on total polyphenol content and antioxidant activity of *Cannabis*. Total phenols data are expressed as equivalent of mg gallic acid/g of extract (mean, n = 3 ± SD). The antioxidant activity data are expressed as % of DPPH inhibition (mean, n = 3 ± SD).

SAMPLE	Extraction Method	Total Phenols	DPPH
CAN1	Maceration with MeOH	111.7 ± 1.1	15.5 ± 0.5
CAN2	Maceration with EtOH	126.4 ± 1	17.4 ± 0.45
CAN3	Soxhlet with MeOH	126.1 ± 1.6	20 ± 0.66
CAN4	UAE with MeOH	145.9 ± 0.9	22.2 ± 0.09
CAN5	SFE CO_2_ at 100 bar, 40 °C	98.8 ± 1	3.4 ± 0.05
CAN6	SFE CO_2_ at 100 bar, 60 °C	38.2 ± 0.6	3 ± 0.02

**Table 3:** Phytocannabinoids in *Cannabis sativa* extracts dissolved in DMSO and analysed by LC-MS/MS. Data are expressed as mg/g (mean, n = 3 ± SD).

Sample	Extraction Method	CBC	CBD	CBDA	CBGA	CBN	THC	THCA
CAN1	Maceration with MeOH	0.669 ± 0.009	17.96 ± 0.12	14.687 ± 0.015	0.279 ± 0.008	0.098 ± 0.001	0.646 ± 0.002	0.022 ± 0.001
CAN2	Maceration with EtOH	0.735 ± 0.005	10.37 ± 0.096	38.948 ± 0.21	0.982 ± 0.068	0.058 ± 0.002	0.582 ±0.003	0.124 ± 0.001
CAN3	Soxhlet with MeOH	0.265 ± 0.009	14.776 ± 0.112	18.542 ± 0.06	0.262 ± 0.009	0.075 ± 0.001	0.353 ± 0.001	0.022 ± 0.001
CAN4	UAE with MeOH	1.825 ± 0.012	5.757 ± 0.009	23.548 ± 0.205	0.537 ± 0.031	0.042 ± 0.002	0.177 ± 0.001	0.055 ± 0.001
CAN5	SFE CO_2_ at 100 bar, 40 °C	0.884 ± 0.01	5.27 ± 0.005	33.769 ± 0.199	0.522 ± 0.012	0.058 ± 0.002	0.362 ± 0.001	0.147 ± 0.002
CAN6	SFE CO_2_ at 100 bar, 60 °C	0.051 ± 0.002	0.238 ± 0.002	2.135 ± 0.005	0.018 ± 0.005	0.007 ± 0.002	0.031 ± 0.001	0.038 ± 0.001

**Table 4:** Growth inhibition IC_50_ values of Caco-2 cells for *Cannabis* extracts and isolates after 48 h (mean ± S.D). Same superscript letters in the table denote no statistical difference and different letters refer to statistically significant difference between results. Full data evaluating statistically significant differences between the samples are shown in Supplementary Materials, Table S6.

SAMPLE	IC_50_ Values (µg/mL) Caco-2
CAN1	12.46 ± 0.35 ^a^
CAN2	8.63 ± 0.54 ^b^
CAN3	13.35 ± 0.51 ^c^
CAN4	12.17 ± 0.72 ^a^
CAN5	14.10 ± 1.17 ^a,c^
CAN6	16.83 ± 2.14 ^c^
THC	14.33 ± 0.79 ^c^
CBD	6.06 ± 0.58 ^d^

**Table 5:** Samples CAN1-CAN6 were prepared using different extraction methods.

Sample	Description
CAN1	Maceration with MeOH
CAN2	Maceration with EtOH
CAN3	Soxhlet with MeOH
CAN4	UAE with MeOH
CAN5	SFE CO_2_ at 100 bar, 40 °C
CAN6	SFE CO_2_ at 100 bar, 60 °C

**Table 6:** Mass spectrometer parameters for LC-MS/MS analysis of cannabinoids.

Component	Ion Precursor	Ion Product	Fragmentation	Collision Energy
CBGA	361	343	100	10
	361	317	100	10
CBDA	359	341	100	10
	359	218.8	100	30
CBD	315.2	193.1	45	20
	315.2	123	45	36
THCA	357.4	313.1	100	10
	357.4	245.1	100	20
THC	311.2	293.2	50	10
	311.2	222.9	50	15
CBN	311.3	293.1	50	16
	311.3	223.1	50	20
CBC	315.3	259.1	100	12
	315.3	81.1	100	15

## Data Availability

The data presented in this study are available within the article and its supplementary material.

## Supplementary Materials

The following are available online at https://www.mdpi.com/2223-7747/10/3/566/s1. Table S1: Solubility of cannabinoids in different solvents expressed as mg/mL (na indicates not applicable, due to lack of literature supported information). Table S2: *p*-values from ANOVA analysis of total phenolic content of various cannabis extracts. Table S3: *p*-values obtained from ANOVA analysis of DPPH activity of various cannabis extracts. Table S4: *p*-values obtained from ANOVA analysis of MTT results in which Caco-2 cells were treated with different cannabis extracts at the concentration 10 µg/mL. Table S5: *p*-values obtained from ANOVA analysis of MTT results in which Caco-2 cells were treated with different cannabis extracts at the concentration 20 µg/mL. Table S6: *p*-values obtained from ANOVA analysis of IC50 in Caco-2 cells. Table S7: *p*-values obtained from ANOVA analysis of MTT results in which HUIEC cells were treated with different cannabis extracts at the concentration 10 µg/mL. Table S8: *p*-values obtained from ANOVA analysis of MTT results in which HUIEC cells were treated with different cannabis extracts at the concentration 20 µg/mL. Table S9: *p*-values obtained from ANOVA analysis of LC/MS-MS results between the crude extracts and DMSO dissolved extracts. Figure S1: A representative LC/MS chromatogram showing a standard cannabinoid used for quantification in cannabis extracts.
