# Supplementary material for: Different Cannabis sativa Extraction Methods Result in Different Biological Activities against a Colon Cancer Cell Line and Healthy Colon Cells
Source: Plants (Basel). 2021 Mar 17;10(3):566. doi: 10.3390/plants10030566 (PMC8002592; doi:10.3390/plants10030566)
Supplement: Supplementary file 1 [file plants-10-00566-s001.pdf]

SUPPLEMENTARY DOCUMENT

Different *Cannabis sativa* Extraction Methods Result in Different Biological Activities Against a Colon Cancer Cell Line and Healthy Colon Cells

Jan Rožanc [1,2,*], Petra Kotnik [3,4], Marko Milojević [1,2], Lidija Gradišnik [1], Maša Knez Hrnčič [3,4], Željko Knez [3,4] and Uroš Maver [1,5,*]

1 University of Maribor, Faculty of Medicine, Institute of Biomedical Sciences, Taborska ulica 8, SI-2000 Maribor, Slovenia; jan.rozanc@um.si, marko.milojevic1@um.si, lidija.gradisnik@um.si
2 BioCore Institute, Nad izviri 8, SI-2204 Miklavž na Dravskem polju, Slovenia; jan.rozanc@biocore.si, marko.milojevic@biocore.si
3 University of Maribor, Faculty of Medicine, Department of Chemistry, Taborska ulica 8, SI-2000 Maribor, Slovenia; petra.kotnik@um.si, zeljko.knez@um.si, masa.knez@um.si
4 University of Maribor, Faculty of Chemistry and Chemical Engineering, Laboratory for Separation processes and Product Design, Smetanova ulica 17, SI-2000 Maribor, Slovenia; petra.kotnik@um.si, zeljko.knez@um.si, masa.knez@um.si
5 University of Maribor, Faculty of Medicine, Department of Pharmacology, Taborska ulica 8, SI-2000 Maribor, Slovenia; uros.maver@um.si

Table S1: Solubility of cannabinoids in different solvents expressed as mg/mL (na indicates not applicable, due to lack of literature supported information).

Solvent	CBC	CBD	CBDA	CBGA	CBN	THC	THCA
MeOH	na	30	na	na	30	30	na
EtOH	30	35	na	30	30	35	35
DMSO	25	60	na	25	50	60	60
DMF	30	50	na	na	50	50	50
DMSO:PBS (pH 7.2) (1:3)	na	0.25	na	na	0.25	0.25	0.25

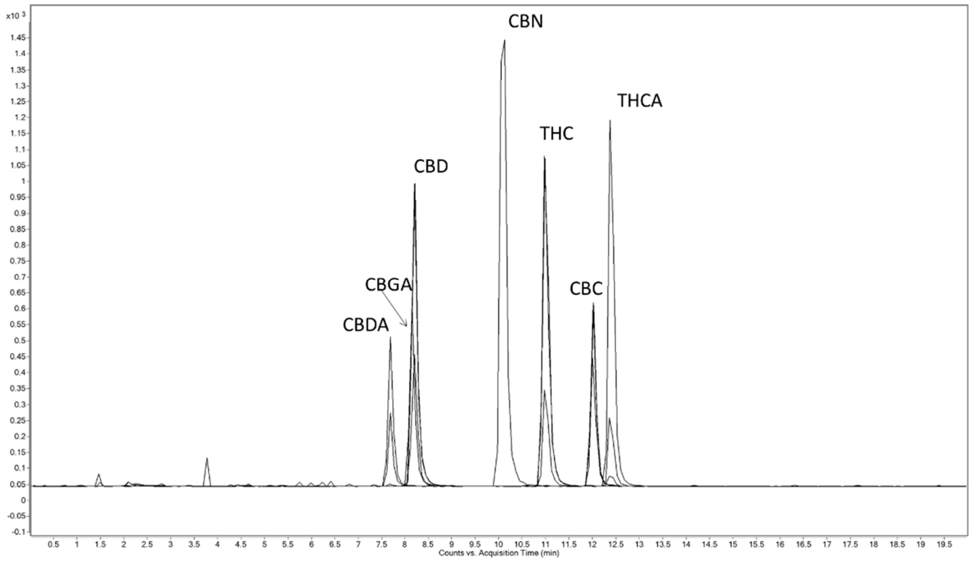

Figure S1: A representative LC/MS chromatogram showing a standard cannabinoid used for quantification in cannabis extracts.

SUPPLEMENTARY DOCUMENT

Table S2: P-values from ANOVA analysis of total phenolic content of various cannabis extracts.

	CAN1	CAN2	CAN3	CAN4	CAN5	CAN6
**CAN1**		6.46E-05	0.000186	2E-06	0.00012	6.07E-08
**CAN2**	6.46E-05		0.785891	1.32E-05	4.3E-06	1.86E-08
**CAN3**	0.000186	0.785891		4.04E-05	1.31E-05	8.01E-08
**CAN4**	2E-06	1.32E-05	4.04E-05		4.48E-07	6.9E-09
**CAN5**	0.00012	4.3E-06	1.31E-05	4.48E-07		9.96E-08
**CAN6**	6.07E-08	1.86E-08	8.01E-08	6.9E-09	9.96E-08	

Table S3: P-values obtained from ANOVA analysis of DPPH activity of various cannabis extracts.

	CAN1	CAN2	CAN3	CAN4	CAN5	CAN6
**CAN1**		0.008994	0.000761	2.7E-05	2.42E-06	2.14E-06
**CAN2**	0.008994		0.004783	5.34E-05	6.97E-07	6.26E-07
**CAN3**	0.000761	0.004783		0.004521	1.62E-06	1.48E-06
**CAN4**	2.7E-05	5.34E-05	0.004521		3.68E-10	3.06E-10
**CAN5**	2.42E-06	6.97E-07	1.62E-06	3.68E-10		5.5E-05
**CAN6**	2.14E-06	6.26E-07	1.48E-06	3.06E-10	5.5E-05	

Table S4: P-values obtained from ANOVA analysis of MTT results in which Caco-2 cells were treated with different cannabis extracts at the concentration 10 μg/mL.

	CAN1	CAN2	CAN3	CAN4	CAN5	CAN6	THC	CBD
**CAN1**		0.000402	0.331682	0.799116	0.282688	0.046737	3.66E-06	3.97E-09
**CAN2**	0.000402		0.000487	0.001749	0.001149	0.000196	6.7E-06	0.000164
**CAN3**	0.331682	0.000487		0.878862	0.577503	0.00957	1.73E-06	8.58E-10
**CAN4**	0.799116	0.001749	0.878862		0.651057	0.192573	0.000117	2.25E-06
**CAN5**	0.282688	0.001149	0.577503	0.651057		0.027816	1.24E-05	1.8E-07
**CAN6**	0.046737	0.000196	0.00957	0.192573	0.027816		1.17E-05	5.86E-09
**THC**	3.66E-06	6.7E-06	1.73E-06	0.000117	1.24E-05	1.17E-05		2.35E-09
**CBD**	3.97E-09	0.000164	8.58E-10	2.25E-06	1.8E-07	5.86E-09	2.35E-09	

Table S5: P-values obtained from ANOVA analysis of MTT results in which Caco-2 cells were treated with different cannabis extracts at the concentration 20 μg/mL.

	CAN1	CAN2	CAN3	CAN4	CAN5	CAN6	THC	CBD
**CAN1**		0.000168	0.000162	3.7E-05	3.68E-05	2.91E-06	0.89108	4.99E-05
**CAN2**	0.000168		0.032662	2.79E-05	2.87E-05	2.4E-06	7.11E-05	0.036916
**CAN3**	0.000162	0.032662		2.53E-05	2.62E-05	2.26E-06	0.000105	0.302419
**CAN4**	3.7E-05	2.79E-05	2.53E-05		0.393354	0.006069	3.71E-05	2.61E-05
**CAN5**	3.68E-05	2.87E-05	2.62E-05	0.393354		0.025135	3.69E-05	2.7E-05
**CAN6**	2.91E-06	2.4E-06	2.26E-06	0.006069	0.025135		2.91E-06	2.3E-06
**THC**	0.89108	7.11E-05	0.000105	3.71E-05	3.69E-05	2.91E-06		2.13E-05
**CBD**	4.99E-05	0.036916	0.302419	2.61E-05	2.7E-05	2.3E-06	2.13E-05	

SUPPLEMENTARY DOCUMENT

Table S6: P-values obtained from ANOVA analysis of IC_{50} in Caco-2 cells.

	CAN1	CAN2	CAN3	CAN4	CAN5	CAN6	THC	CBD
**CAN1**		7.73E-05	0.0342	0.494018	0.401756	0.032159	0.012382	7.75E-06
**CAN2**	7.73E-05		1.5E-05	0.000247	0.002482	0.005637	7.72E-05	0.000569
**CAN3**	0.0342	1.5E-05		0.042821	0.643519	0.06085	0.089506	1.42E-06
**CAN4**	0.494018	0.000247	0.042821		0.259533	0.017416	0.006649	1.19E-05
**CAN5**	0.401756	0.002482	0.643519	0.259533		0.033509	0.123032	0.000433
**CAN6**	0.032159	0.005637	0.06085	0.017416	0.033509		0.121766	0.002491
**THC**	0.012382	7.72E-05	0.089506	0.006649	0.123032	0.121766		2.84E-06
**CBD**	7.75E-06	0.000569	1.42E-06	1.19E-05	0.000433	0.002491	2.84E-06	

Table S7: P-values obtained from ANOVA analysis of MTT results in which HUIEC cells were treated with different cannabis extracts at the concentration 10 μg/mL.

	CAN1	CAN2	CAN3	CAN4	CAN5	CAN6	THC	CBD
**CAN1**		0.000937	0.000515	0.021332	0.000815	0.021954	0.906755	0.125850
**CAN2**	0.000937		0.788413	0.004112	0.944244	0.007353	0.000683	0.000033
**CAN3**	0.000515	0.788413		0.000980	0.842731	0.002338	0.000359	0.000010
**CAN4**	0.021332	0.004112	0.000980		0.003066	0.889354	0.015246	0.000445
**CAN5**	0.000815	0.944244	0.842731	0.003066		0.005750	0.000589	0.000026
**CAN6**	0.021954	0.007353	0.002338	0.889354	0.005750		0.015999	0.000601
**THC**	0.906755	0.000683	0.000359	0.015246	0.000589	0.015999		0.142463
**CBD**	0.125850	0.000033	0.000010	0.000445	0.000026	0.000601	0.142463	

Table S8: P-values obtained from ANOVA analysis of MTT results in which HUIEC cells were treated with different cannabis extracts at the concentration 20 μg/mL.

	CAN1	CAN2	CAN3	CAN4	CAN5	CAN6	THC	CBD
**CAN1**		0.374828	0.42661	0.005398	0.022315	0.007679	7.33E-11	3.79E-07
**CAN2**	0.374828		0.904657	0.006393	0.312854	0.017245	2E-08	1.21E-06
**CAN3**	0.42661	0.904657		0.01138	0.501651	0.038943	1.45E-07	3.25E-06
**CAN4**	0.005398	0.006393	0.01138		0.00143	0.095552	2.17E-07	9.75E-06
**CAN5**	0.022315	0.312854	0.501651	0.00143		0.001274	1.57E-09	4.22E-07
**CAN6**	0.007679	0.017245	0.038943	0.095552	0.001274		1.84E-09	1.06E-06
**THC**	7.33E-11	2E-08	1.45E-07	2.17E-07	1.57E-09	1.84E-09		0.000205
**CBD**	3.79E-07	1.21E-06	3.25E-06	9.75E-06	4.22E-07	1.06E-06	0.000205	

Table S9: P-values obtained from ANOVA analysis of LC/MS-MS results between the crude extracts and DMSO dissolved extracts.

SAMPLE	Extraction method	CBC	CBD	CBDA	CBGA	CBN	THC	THCA
CAN1	Maceration - MeOH	0,000849	5,23E-05	4,39E-05	2,59E-09	0,000105	0,000287	2,23E-05
CAN2	Maceration - EtOH	0,000353	1,16E-09	1,94E-06	0,000107	1,45E-07	0,000227	1,15E-05
CAN3	Soxhlet - MeOH	0,000198	6,75E-08	4,03E-05	0,00046	7,3E-07	0,000268	4,44E-06
CAN4	UAE - MeOH	0,028545	6,92E-06	8,56E-05	0,000499	8,14E-06	0,000299	4,37E-06
CAN5	SFE 100 bar, 40 °C	0,000479	2,2E-05	0,00012	0,001143	2E-05	0,00019	5,39E-06
CAN6	SFE 100 bar, 60 °C	0,004902	8,37E-06	2,15E-05	2,32E-07	0,000389	0,000333	8,13E-06
